# *PDB2INS*: bridging the gap between small-molecule and macromolecular refinement

**DOI:** 10.1107/S1600576719005478

**Published:** 2019-05-14

**Authors:** Anna V. Lübben, George M. Sheldrick

**Affiliations:** a Abteilung für Strukturchemie, Universität Göttingen, Tammannstrase 4, Göttingen, D-37077, Germany

**Keywords:** crystal structure refinement, macromolecules, PDB format, *SHELXL*

## Abstract

The open-source Python program *PDB2INS* is designed to prepare a .ins file for refinement with *SHELXL* [Sheldrick (2015). *Acta Cryst.* C**71**, 3–8], taking atom coordinates and other information from a Protein Data Bank (PDB)-format file. If *PDB2INS* is provided with a four-character PDB code, both the PDB file and the accompanying mmCIF-format reflection data file (if available) are accessed via the internet from the PDB public archive [Read *et al.* (2011). *Structure*, **19**, 1395–1412] or optionally from the *PDB_REDO* server [Joosten, Long, Murshudov & Perrakis (2014). *IUCrJ*, **1**, 213–220]. The *SHELX*-format .ins (refinement instructions and atomic coordinates) and .hkl (reflection data) files can then be generated without further user intervention, appropriate restraints *etc*. being added automatically. *PDB2INS* was tested on the 23 974 X-ray structures deposited in the PDB between 2008 and 2018 that included reflection data to 1.7 Å or better resolution in a recognizable format. After creating the two input files for *SHELXL* without user intervention, ten cycles of conjugate-gradient least-squares refinement were performed. For 96% of these structures *PDB2INS* and *SHELXL* completed successfully without error messages.

## Abstract 2

*PDB2INS* reads a Protein Data Bank (PDB)-format file and writes a .ins file containing the atom data and instructions for *SHELXL* refinement. Given only the PDB code of a deposited structure, it automatically creates both files (.ins and .hkl) necessary to refine the structure.

## 1. Introduction   

Historically, computer methods for crystal structure refinement developed relatively independently for inorganic, organometallic and organic structures on the one hand and biological macromolecules on the other. This resulted in many incompatibilities involving file formats and nomenclature. *SHELXL* (Sheldrick, 2015 ▸; http://shelx.uni-goettingen.de/), probably the most widely used program for small-molecule refinement, has some features – *e.g.* the estimation of least-squares standard deviations for the refined parameters, the ease of handling complicated disorders and non-merohedral twins, and the powerful concept of ‘free variables’ – that might be useful for macromolecular refinement when high-resolution data are available. For an example of a macromolecular refinement in which the estimation of least-squares standard deviations played a decisive role see Köpfer *et al.* (2014) ▸. *SHELX* was written for small-molecule refinement in the early 1970s. Major extensions in the 1990s (*e.g.* the introduction of residues and the removal of restrictions on the number of atoms) first made it possible to use it for macromolecular refinement (Sheldrick, 1993 ▸; Sheldrick & Schneider, 1997 ▸), but the extensive reformatting required was still an impediment. The adoption of CIF format might have simplified this problem, but unfortunately mmCIF and small-molecule CIF are hardly compatible; for example, even the unit-cell dimensions have different names.

The original implementation of the least-squares refinement in *SHELXL* was based closely on a scheme proposed by Cruickshank (1969 ▸). *SHELXL* uses a conventional structure factor summation rather than a fast Fourier transform and implements the calculation of *R*_complete_ (Luebben & Gruene, 2015 ▸) as well as *R*_free_ (Brünger, 1992 ▸). It offers a choice of full- or blocked-matrix refinement or conjugate-gradient solution of the least-squares normal equations (Konnert & Hendrickson, 1980 ▸). It may be used for crystal structure refinement against single-wavelength or Laue X-ray, neutron or electron diffraction data (Gruene *et al.*, 2014 ▸; Clabbers *et al.*, 2019 ▸) and can also handle merohedral and non-merohedral twins.

The Python program *PDB2INS* is designed to automate setting up a *SHELXL* refinement starting from a macromolecular structure in PDB format (http://www.rcsb.org/, https://pdbj.org/, https://www.ebi.ac.uk/pdbe/ and https://pdb-redo.eu/). It replaces the Fortran program *SHELXPRO* (Sheldrick & Schneider, 1997 ▸) that was originally distributed with *SHELX* for this purpose.

## 2. Input files for *SHELXL*   

If *PDB2INS* is provided with a four-character PDB code, both the PDB file and the accompanying mmCIF-format reflection data file (if available) are accessed via the internet from the PDB public archive (Read *et al.*, 2011 ▸) or optionally from the *PDB_REDO* server (Joosten *et al.*, 2014 ▸). The *SHELX*-format .ins (refinement instructions and atomic coordinates) and .hkl (reflection data) files can then be generated without further user intervention, appropriate restraints *etc*. being added automatically.

**Figure 1:**
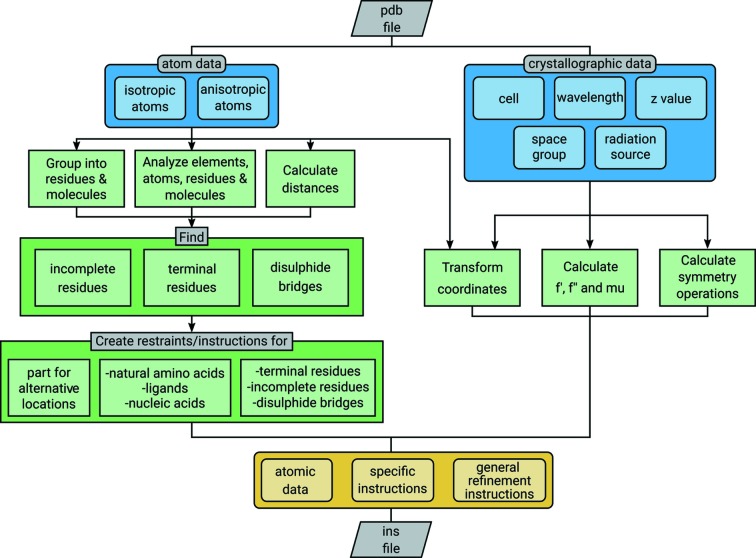
Flowchart illustrating the organization of *PDB2INS*.

A flowchart llustrating the organization of *PDB2INS* is shown in Fig. 1 ▸.

### 2.1. Representation of atoms and residues   

An atom in a PDB file (Dutta *et al.*, 2009 ▸) must have a unique combination of chain identifier (one character), residue sequence number (up to four digits), alternative location character and atom name (up to four characters), and in addition it has a residue name (up to three characters) and may have a one-character insertion code. Residue names and numbers considerably simplify the application of restraints in *SHELXL* refinements, also for small molecules, because the same names can be used for atoms in similar residues. For example, the instruction

FLAT­_TOL C1 > C7

could be used to restrain the carbon atoms in each toluene residue to lie in a plane. The original small-molecule approach of using different names for each atom would involve much more typing and is less intuitive and more error prone. Residue names and numbers are set by RESI instructions such as

RESI TOL 21

which would be followed by a residue consisting of one toluene molecule. Such RESI instructions remain in force until the next RESI instruction is read. Several residues with different residue numbers may have the same residue name but not *vice versa*. Residue numbers for *SHELXL* must be between −999 and 9999 inclusive. Chain identifiers were needed for compatibility with the PDB but were first introduced into *SHELXL* in 2016. This required a major reorganization of the code and caused incompatibilities with several legacy programs. Chain identifiers are required for very large structures but can be useful, even for small molecules, when there are several similar molecules in the asymmetric unit. The residue numbers in the RESI instructions are then extended to include the chain IDs, *e.g.*

RESI TOL A:21

for residue 21 in chain A. For *SHELX* the chain identifiers may only be upper- or lower-case letters, digits, or the blank character, so there are only 63 different possible chain identifiers. This is enough except for the very largest structures in the PDB. Note that chain identifiers are an exception to the usual *SHELX* rule that upper- and lower-case letters are treated as equivalent.

### 2.2. Treatment of symmetry and transformations of coordinates and displacement parameters   

*SHELXL* expects the space-group symmetry to be defined by the coordinates of the general position rather than the space-group name. This permits the use of non-standard settings. *PDB2INS* uses a dictionary approach in which the symmetry generators (Fischer & Koch, 2005 ▸) are stored for each non-centrosymmetric space-group symbol and used to generate the *SHELX*-format SYMM instructions by iterative multiplication. The open-source Python module SPAGSYDATA used by *PDB2INS* to do this is available from https://github.com/av-luebben/spagsydata.

For example, the space group *R*3 is defined by

LATT -3

SYMM -Y, X-Y, Z

SYMM -X+Y, -X, Z

on hexagonal axes or

LATT -1

SYMM Z, X, Y

SYMM Y, Z, X

on primitive rhombohedral axes.

*SHELXL* refines fractional coordinates rather than the Cartesian coordinates used in the PDB, so *PDB2INS* applies the appropriate transformations to the atomic coordinates and atomic displacement parameters.

### 2.3. Hydrogen atoms   

*PDB2INS* includes HFIX instructions in the .ins file for generating hydrogen atoms in the form of comments, each prefaced with REM, *e.g.* for a valine residue

REM HFIX_VAL 43 N

REM HFIX_VAL 13 CA CB

REM HFIX_VAL 33 CG1 CG2

Later in the refinement the user can delete ‘REM ’ to activate the hydrogen-atom generation. Such riding hydrogen atoms do not change the number of parameters refined. So that missing atoms in a side chain do not cause an error when *SHELXL* later tries to generate hydrogen atoms, *PDB2INS* adds ‘HFIX 0’ instructions that can be edited when the structure becomes more complete.

### 2.4. Geometrical restraints   

*PDB2INS* automatically adds the restraints on 1,2- and 1,3-distances corresponding to the restraints on bond distances and angles in amino acids given by Engh & Huber (1991 ▸). In addition *PDB2INS* uses a library of restraints for other common residues that were generated using the *Grade* server (http://grade.globalphasing.org/). Alternatively, suitable geometrical restraints can be generated by *PROSMART* (Nicholls *et al.*, 2012 ▸). *PDB2INS* uses interatomic distances to detect disulfide bridges and C- and N-terminal residues and adds appropriate geometrical restraints and HFIX instructions. *SHELXL* uses planarity restraints rather than torsion angle restraints to ensure peptide planarity. A side effect of this is that it is possible for a *trans*-peptide to refine with *SHELXL* to *cis* or *vice versa* if the data strongly indicate that this is required (Stenkamp, 2005 ▸)

### 2.5. Restraints on atomic displacement parameters   

Anisotropic refinement requires six parameters per atom instead of one for an isotropic refinement. This is too many for most macromolecules, although the rigid-bond restraint DELU may be used to make the motions of bonded atoms along the bond joining them more equal, and the SIMU restraint to make the *U_ij_* anisotropic displacement parameters of two atoms more equal is particularly useful for disordered models in which atoms overlap. However, the more recent RIGU extended rigid-bond model (Thorn *et al.*, 2012 ▸) leads to a substantial reduction in the number of effective parameters. The RIGU model simply assumes that the relative motion of two bonded atoms is at right angles to the bond joining them, reducing the effective number of parameters per atom to three. Applying RIGU to 1,3-distances leads to a further reduction. An additional constraint (XNPD) imposes a minimum value for the motion of an atom in any direction, preventing displacement ellipsoids from becoming non-positive definite. Taken together, these two options that can be applied globally with the instructions RIGU and XNPD 0.01 enable structures to be refined anisotropically at appreciably lower resolution than previously possible. *PDB2INS* always writes these instructions to the .ins file, but RIGU only takes effect when the atoms are made anisotropic with the instruction ANIS.

### 2.6. Wavelength-specific considerations   

*SHELXL* stores the scattering factors of the first 98 elements in the periodic table, recognizes the wavelengths of the more common in-house sources (Ga, Cu, Mo, Ag and In), and sets the absorption and dispersion coefficients for them automatically. *PDB2INS* sets up DISP instructions giving the values of *f*′, *f*′′ and μ generated automatically from Kissel tables (Roy *et al.*, 1993 ▸) using the given wavelength. For neutron diffraction, the user must insert a NEUT instruction. For naturally occurring elements, the average isotopic distribution is assumed for the scattering lengths; for synthetic isotopes, the most common isotope is assumed. For other isotopes, the user must insert the appropriate SFAC instruction. Starting with *SHELXL2019*/*1*, if the wavelength is shorter than 0.1 Å the reflection data are assumed to be electron diffraction data and electron scattering factors generated using the Mott–Bethe formula are used. In all cases the scattering factors and dispersion corrections may be set by hand by editing the .ins file to include appropriate SFAC instructions.

### 2.7. Reflection data   

The *SHELX* reflection data file (.hkl) was originally designed for 80-column punched cards. There is one reflection per line in this fixed-format text file, with the structure

HHHHKKKKLLLLRRRRRRRRSSSSSSSSBBBB

The reflection indices *h*, *k*, *l* are right-justified integers. For the intensities R and their standard deviations S, the position of the decimal point determines how these floating-point numbers are read. If the decimal point is missing these numbers are read as right-justified integers and then converted to floating point. If the .ins file ends with HKLF 4, R and S are intensities and their standard deviations; if it ends in HKLF 3, they are *F* and σ(*F*). The four characters B were historically the batch number (*e.g.* for Weissenberg films). Now this is normally −1 for a free-*R* reflection and +1 or absent for the reflections used for refinement. The program *mtz2hkl* (Grune, 2008 ▸) may be used to convert a CCP4 .mtz reflection data file to *SHELX* .hkl format.

### 2.8. Python implementation and program availability   

*PDB2INS* is written in object-oriented Python 2.7. *PyInstaller* (http://www.pyinstaller.org/) was used to compile it into stand-alone executables. *PDB2INS* is open source and may be downloaded as part of CCP4 (Winn *et al.*, 2014 ▸; http://www.ccp4.ac.uk/), from the *SHELX* server (http://shelx.uni-goettingen.de/) or from Git-Hub (https://github.com/av-luebben/pdb2ins), where the code was published. In addition to the command-line version, a free graphical user interface (GUI) version that is designed to help inexperienced users may be downloaded from https://github.com/av-luebben/PDB2INSGUI or from the *SHELX* server. *PDB2INS* is available for 64-bit Linux, MacOSX and Windows systems. The GUI was written using TkInter (Tcl/Tk), the *de facto* standard graphical interface for Python (https://wiki.python.org/moin/TkInter).

Further information on using *PDB2INS* may be obtained by typing PDB2INS --help. After preparing the input files with the help of *PDB2INS*, *SHELXL* is usually run from the command line, *e.g.*

shelxl name

which reads the .ins and .hkl input files. This produces a listing file .lst and an updated instruction and structure file .res, and optionally a PDB-format file .pdb and a .fcf file containing observed and calculated structure factors. *Coot* (Emsley *et al.*, 2010 ▸) may be used to inspect the results of the refinement. The .res file is copied to a .ins file for the next refinement job; often it will be necessary to edit it to include additional disorder components specified by PART numbers *etc*.

## 3. Test results   

*PDB2INS* was tested on 23 974 data sets deposited between 2008 and 2018 in the PDB with a resolution of 1.7 Å or better. Only 4.0% (964 data sets) displayed any problems. Details are given in Table 1 ▸. For the remaining 96% the *SHELXL* refinement was successful without needing to make any changes to the .ins and .hkl files written by *PDB2INS*. Potential problems caused by insertion codes in the PDB file were avoided by renumbering the residues. In general the *R* factors obtained using *SHELXL* tend to be slightly higher than those obtained using *Refmac* (Murshudov *et al.*, 2011 ▸) or *Phenix* (Adams *et al.*, 2010 ▸), mainly as a result of the less sophisticated Babinet bulk solvent model (Moews & Kretsinger, 1975 ▸) still employed by *SHELXL*.

## Figures and Tables

**Table 1:** Overview of the most common issues detected during an automated test of *PDB2INS* against a sample of 23 974 data sets from the PDB with a resolution of 1.7 Å or better Only 4.02% (964 data sets) displayed any problems. %_E_ percentage related to all errors/issues, %_T_ percentage based on the complete test set.

%_E_	%_T_	Issue description
31.74	1.28	*SHELXL* terminated without refinement because the residue has a name consisting only of digits. This is allowed in the PDB but not in *SHELXL* because it could be mistaken for a residue number. *PDB2INS* will prompt the user to change such residue names when run in interactive mode. However in automated mode, *PDB2INS* only writes a warning and continues without renaming.
22.41	0.90	One or more reflections do not adhere to the required .hkl format.
11.20	0.45	An element name in the PDB file does not correspond to any of the first 98 elements of the periodic table as required by *SHEXL*. Most commonly it has been specified as ‘X’.
10.68	0.43	Warning: one or more atoms are not subject to appropriate restraints. Of course for heavy atoms or high-resolution data this may be intended.
10.37	0.42	A reflection data mmCIF file is incomplete, *e.g.* because standard uncertainties are missing or because *I*+ but not *I*− was specified.
4.67	0.19	It is legal to deposit more than one model in the same .pdb file. *PDB2INS* cannot handle such files.
8.93	0.35	A variety of other issues can occur, but each in less than 0.1% of all tested files. The error messages output by *PDB2INS* and *SHELXL* should normally enable the problems to be identified.
